# Effectiveness of Using a Digital Wearable Plantar Pressure Device to Detect Muscle Fatigue: Within-Subject, Repeated Measures Experimental Design

**DOI:** 10.2196/65578

**Published:** 2025-01-07

**Authors:** Fu-Yu Chen, Tzu-Yao Lin, Yi-Cheng Huang, Evina Widianawati

**Affiliations:** 1 Department of Biomedical Engineering, Chung Yuan Christian University, No. 200, Zhongbei Road, Zhongli District, Toayuan City, 32023, Taiwan, 886 32564507

**Keywords:** muscle fatigue, plantar pressure sensors, wearable devices, home-based monitoring

## Abstract

**Background:**

Muscle fatigue, characterized by reduced force generation during repetitive contractions, impacts older adults doing daily activities and athletes during sports activities. While various sensors detect muscle fatigue via muscle activity, biochemical markers, and kinematic parameters, a real-time wearable solution with high usability remains limited. Plantar pressure monitoring detects muscle fatigue through foot loading changes, seamlessly integrating into footwear to improve the usability and compliance for home-based monitoring.

**Objective:**

This study aimed to investigate the effects of muscle fatigue on plantar pressure measurements using a self-developed wearable plantar pressure system.

**Methods:**

Twelve healthy participants completed a 5-minute calf muscle fatigue protocol. The plantar pressures and surface electromyography (sEMG) activity of the gastrocnemius muscles were recorded before and after exercise. The plantar pressures at 6 regions and the median frequency (MDF) of sEMG were analyzed to quantify fatigue.

**Results:**

The self-developed foot pressure system showed a significant decrease in plantar pressure peak values at the heel of the left (*P*=.003) and right feet (*P*=.001) and at the lateral toe of the left (*P*=.001) and right feet (*P*=.026). A significant increase was observed at the metatarsal head of both the left foot (*P*=.001) and the right foot (*P*=.017). The MDF of sEMG signals significantly decreased in the left (*P*=.001) and right gastrocnemius (*P*<.001).

**Conclusions:**

Plantar pressure changes and sEMG signals effectively detect gastrocnemius muscle fatigue using the proposed wearable system, supporting the development of a wearable solution for detecting muscle fatigue suitable for home-use.

## Introduction

Muscle fatigue, characterized by the reduced ability to generate adequate force during repetitive contractions [1], affects performance across different groups and activities including daily activities of older adults as well as athletes during sports. Muscle fatigue disrupts the neuromuscular system and affects different aspects such as muscle strength, force production, and movement patterns, resulting in stiffness of the muscles and joints, impaired motor control, and poor balance [2], thereby increasing the risk of injuries. For instance, in badminton players, muscle fatigue impairs neuromuscular coordination, by reducing input from the foot sole receptors, and muscle force production, leading to weaker responses and decreased reaching distance performance [3]. Running-induced fatigue reduces lower limb muscle activity, decreases shock absorption capacity, and alters plantar pressure distribution [4], thus increasing the risk of running-related injuries such as stress fractures in the foot [5]. Mello et al highlighted how fatigue delays the gastrocnemius muscle’s activation by 1 second relative to the center of pressure, thus impairing balance [6]. Furthermore, Morrison et al found that muscle fatigue in older adults (aged 60‐79 years) significantly increased reaction times, postural sway, and fall risk compared to younger individuals (aged 30‐59 years) [7].

Muscle fatigue can be detected through various methods, including blood lactate concentration [8], electromyography (EMG) [9], mechanomyography (MMG) [10], near-infrared spectroscopy (NIRS) [11], and kinematic parameters using inertial measurement units (IMUs) [12]. While the lactate concentration provides an estimation of global fatigue, it cannot monitor fatigue in real-time [8]. MMG, NIRS, and IMUs can be used in wearable forms; however, the use of NIRS and MMG has challenges owing to issues such as time lags, the use of MMG has susceptibility to motion artifacts [13], and the use of IMUs needs further research regarding the relationship between kinematic parameters and muscle fatigue [14]. Surface electromyography (sEMG) is a widely used wearable method for real-time monitoring of local muscle fatigue by measuring myoelectric activity [15]. Fatigue reduces muscle fiber conduction velocity, shifting the sEMG power spectrum to lower frequencies (eg, decreased median frequency) and increasing signal amplitude. sEMG captures these changes and quantifies muscle fatigue using time-domain or spectral parameters [8]. However, the use of sEMG in a home setting is difficult, owing to the challenges of correctly attaching the electrodes and regularly wearing the device without assistance [16].

Adherence is a critical factor for effective daily monitoring, as meaningful and continuous data are required for accurate analysis [17]. Wearable devices integrated with mobile apps present promising healthcare solutions for home-based monitoring. The integration of hardware sensors and software mobile apps enables continuous and unobtrusive monitoring, thereby providing real-time data analysis that can support timely interventions [18]. A suitable alternative wearable approach for muscle fatigue monitoring is the use of plantar pressure sensors, which can be seamlessly integrated into a shoe’s insole for daily comfortable wear. Wearable plantar pressure sensors in the foot insole are widely used for the detection of diabetic foot ulcers [19], gait analysis, and the measurement of the ground reaction force [20] and the center of pressure [6]. Muscle fatigue impacts body mechanics and alters foot loading patterns, measurable through plantar pressure at different foot regions. Considerable research has explored changes in plantar pressure following various physical activities linked to lower limb muscle fatigue, such as badminton [21], walking [22], and long-distance walking [23].

Despite the aforementioned advancements, wearable foot plantar systems for detecting muscle fatigue in everyday physical activities have yet to be designed. In this study, the effect of calf muscle fatigue in healthy participants was investigated using a self-developed wearable plantar pressure system that was suitable for daily use. The results can provide a muscle fatigue detection method for developing a wearable plantar pressure monitoring system

## Methods

### The Digital Foot Pressure System

**Figure 1.**
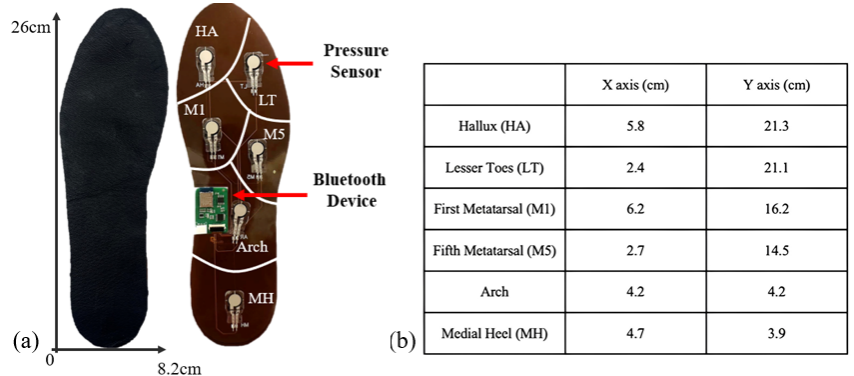
Wearable plantar pressure device in an insole format. (**A**) Foot insole with 6 regions of pressure measurements: hallux (HA), lesser toe (LT), first metatarsal (M1), fifth metatarsal (M5), arch, and medial heel (MH); (**B**) Coordinates of the 6 pressure sensors on the insole.

**Figure 2.**
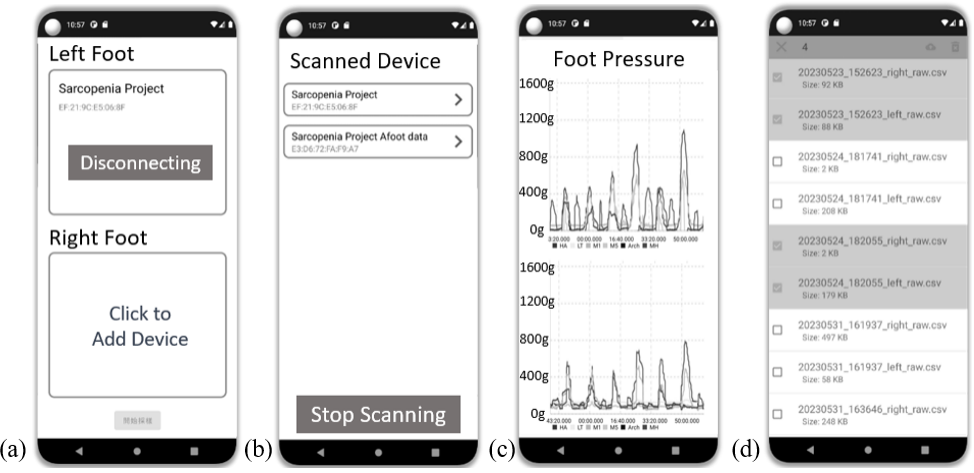
Overview of the digital mobile app for the wearable plantar pressure device. (**A**) Bluetooth connection page for the devices on the right and left feet; (**B**) Bluetooth connectable device page; (**C**) real-time streaming graph showing plantar pressure data; and (**D**) Cloud storage for historical data.

A self-developed digital foot pressure system was utilized in this study. Figure 1 shows the wearable plantar devices for both the left and right feet as well as the measurements of the devices. Each device, designed to be worn comfortably with shoes, is equipped with 6 pressure sensors (A301 Flexiforce, Tekscan Inc.). The system also has a nRF52840 microprocessor (Nordic Semiconductor) used for the analog-to-digital converter measurement of foot plantar pressure, a wireless Bluetooth 5.0 module for data transmission, and a lithium-ion battery for power supply. The system operates with a sampling rate of 100 Hz, ensuring high-resolution data capture. The data from the 6 pressure sensors in both the left and right devices can be transmitted wirelessly in real time via Bluetooth to a custom-developed Android mobile app. This app facilitates real-time data display, analysis, and cloud storage, as shown in Figure 2. The pressure sensors are strategically arranged on the foot insole to cover 4 main plantar anatomical areas: the toes, metatarsals, arch, and heel. This general layout enables accurate measurement of natural gait; the layouts of the toes and metatarsals are further divided into 2 parts to capture plantar pressures along the mediolateral axis during walking [24].

### Ethical Considerations

The research was approved by the Ethics Review Board of the Chung Yuan Christian University for Human Subjects Research (No. 8800-4-07-002). The participants were informed of the requirements and procedures of the entire experiment, and written consent was obtained before testing.

### Subjects

A briefing session was conducted to familiarize the participants with the fatigue protocol and the walking test to be used in the research. Twelve healthy participants from the Chung Yuan Christian University, Taiwan, were recruited for the research. The mean (SD) age of the participants was 23.7 (2.6) years, and their mean (SD) weight was 62.4 (6.3) kg. The dominant limb of each participant was determined by identifying which foot would step out first once they started walking. The exclusion criteria included participants with plantar fasciitis or flatfoot and those who could not run independently for 5 minutes. Given that these foot disorders were excluded, experiments for minimizing variability could be performed. Then, suitable foot insole sizes were given to the participants.

### Experimental Procedures

The experimental procedures were divided into 4 main steps. First, baseline tests were performed to measure the EMG of muscle activity and foot plantar pressure in both the legs. During the baseline test, the sEMG activities of the participants’ gastrocnemius and rectus femoris muscles in the right and left legs were recorded using sEMG electrodes (T709, Comepa, France) and a data acquisition instrument (MP36, Biopac Inc., USA). The skin of each participant was cleaned with alcohol before the electrodes were attached. The sEMG muscle activities were recorded at a sampling rate of 2000 Hz. The plantar pressures at 6 locations in the left and right legs were measured simultaneously with sEMG. The plantar pressures were recorded using the wearable foot plantar device that was developed specifically for this study. Then, timed up-and-go and 10-meter walk tests were conducted; the participants were asked to rise from a standard chair, walk for 10 meters at their most comfortable speed, turn around, walk back. and sit down again [25].

After the baseline test, each participant was instructed to perform a fatigue exercise. All the participants were asked to place a weight-bearing apparatus on each leg and perform forefoot running for 5 minutes. The weight-bearing apparatus was 1/20th of a participant’s weight [26]. sEMG data were collected from the gastrocnemius and rectus femoris muscles to capture the electrical activity associated with muscle contractions, and the wearable foot plantar pressure device was used to monitor changes in pressure distribution under the feet. This dual approach allowed the fatigue levels in both the muscles to be evaluated and quantified. After the exercise, the rating of perceived exertion (RPE) scale was used to assess and document each participant’s perceived level of fatigue. Postfatigue measurements were recorded immediately after the fatigue protocol, while the participants performed timed up-and-go and 10-meter walk tests, and sEMG and foot plantar pressure measurements for both legs were collected.

### EMG Muscle Fatigue Analysis

Three consecutive sEMG signals from the gastrocnemius and rectus femoris muscles were averaged for prefatigue and postfatigue analyses. The sEMG envelope was calculated from the raw sEMG signal through a 20‐500–Hz finite impulse response bandpass filter. The signal was then processed via fast Fourier transformation to calculate the power spectrum median frequency (MDF) of the sEMG. A shift in the MDF to a lower frequency during exercise is considered localized muscle fatigue [27]. This method allowed researchers to determine whether muscle fatigue was induced in the gastrocnemius muscles, thereby increasing confidence in these data’s correlation with foot plantar pressure data. The MDF is the frequency at which the EMG power spectrum is transmitted, and it is given by


(1) $$\sum_{j=1}^{MDF}\;P_{j}=\sum_{j=MDF}^{M}\;P_{j}=\frac{1}{2}\sum_{j=1}^{M}\;p_{j},$$

where $P_{j}$ is the power spectrum of the EMG at frequency bin *j*. The MDF splits the power spectrum of the EMG into two equal-amplitude regions.

### The RPE Scale Analysis

The RPE scale was utilized to assess each participant’s self-reported fatigue level. This subjective measurement tool is widely employed in the fields of physical activity, exercise, and sports to evaluate an individual’s perceived effort and fatigue during physical tasks [28]. Participants rate their exertion based on sensations such as increased heart rate, breathing rate, muscle fatigue, and overall physical strain. The Borg RPE Scale, ranging from 6 to 20, is one of the most commonly used versions, with higher scores indicating greater levels of exertion [29].

### Foot Plantar Pressure Analysis

Foot plantar pressure data, obtained using the developed digital foot plantar system and the commercial system, were simultaneously collected with the EMG data. The 6 plantar pressures in the left and right feet obtained in this research represent 6 anatomical areas of the foot: hallux (HA), lesser toe (LT), first metatarsal (M1), fifth metatarsal (M5), arch, and medial heel (MH). The peak pressures (Pa) in these 6 anatomical areas were analyzed and calculated from the raw plantar pressure data via MATLAB version R2022a (Mathworks Inc.). The measurements for the three consecutive prefatigue and postfatigue tests were averaged and compared. On the basis of the preliminary results, changes in the mean peak pressure of the 6 anatomical areas before and after the fatigue protocol were determined.

### Statistical Analysis

In this study, the MDFs of the sEMG signals and the mean peak plantar pressures in each anatomical area for each participant before and after the fatigue protocol are presented as the means (standard errors). A paired *t* test with repeated measures was used to compare the MDFs of sEMG signals from the gastrocnemius and rectus femoris muscles before and after fatigue exercise. Then, a paired *t* test with repeated measures was used to detect significant differences in the 6 anatomical areas (ie, HA, LT, M1, M5, arch, and MH) before and after muscle fatigue exercise for the wearable plantar pressure system. All the statistical tests were performed with the significance level of *P*<.05.

## Results

The data for muscle fatigue, RPE, and mean plantar pressure were collected and analyzed using a paired *t* test.

### MDFs of sEMG in Detecting Muscle Fatigue

**Figure 3.**
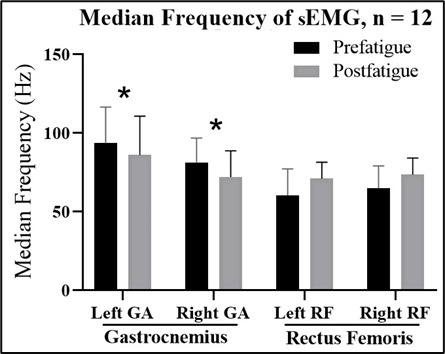
Medium frequency of the sEMG for the gastrocnemius (GA) and rectus femoris (RF) muscles in the left and right legs before the fatigue exercise (prefatigue) and after the fatigue exercise (postfatigue). sEMG data of the left and right gastrocnemius muscles significantly decreased after the exercise for muscle fatigue (*P*=.001 and *P*<.001, respectively).

The MDFs of the sEMG data of the gastrocnemius and rectus femoris muscles are presented in Figure 3. The MDFs (SD) of the sEMG data of the left and right gastrocnemius muscles significantly decreased from 93.6 (22.8) Hz to 86.1 (24.6) Hz in the left foot and from 81.3 (15.5) Hz to 72.1 (16.6) Hz in the right foot after exercise for muscle fatigue (*P*=.001 and *P*<.001, respectively). However, the MDF of the sEMG before and after muscle fatigue exercise did not significantly differ between the left and right rectus femoris muscles.

### The RPE Scale in the Muscle Fatigue Protocol

The RPE scale was used in the experiment to assess the muscle fatigue levels of the 12 participants. All the participants reported that the level of fatigue in the gastrocnemius muscle was greater than that in the rectus femoris. The analysis of the average RPE scores revealed that the mean fatigue level for the gastrocnemius muscle was 14.3 (SD 1.3), whereas the mean fatigue level for the rectus femoris was 7.0 (SD 1.3). The significant difference in fatigue levels (P <.001) between the two muscles aligns with the analysis of MDF in the sEMG data. These findings confirmed that the gastrocnemius muscle experienced greater fatigue than the rectus femoris did during the experiment.

### Foot Plantar Pressure in Detecting Muscle Fatigue

**Figure 4.**
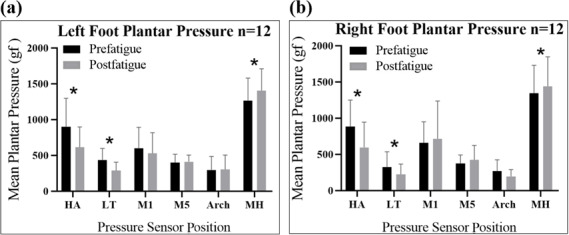
Mean peak plantar pressure before the fatigue exercise (prefatigue) and after the fatigue exercise (postfatigue) by sensor positions: hallux (HA), lesser toe (LT), first metatarsal (M1), fifth metatarsal (M5), arch, and medial heel (MH) in the (**A**) left foot: a statistically significant decrease in the mean plantar pressure peak values was observed in the HA (*P*=.003) and LT (*P*=.001), while the MH showed a statistically significant increase (*P*=.001) after the fatigue exercise; and (**B**) right foot: a statistically significant decrease in the mean plantar pressure peak values was observed in the HA (*P*=.001) and LT (*P*=.026). A significant increase was noted in the MH position after fatigue (*P*=.017).

The data for the left and right feet (Figure 4A and B, respectively) were obtained using a developed digital plantar pressure measurement system. Figure 4 shows the changes in plantar pressure before and after fatigue. The changes were noticeable in terms of the plantar pressure at the HA, LT, and MH on both feet after gastrocnemius muscle fatigue. The pressure values at other locations also changed, but they were not statistically significant.

The mean plantar pressure values were measured using the self-developed foot pressure system. A significant decrease was observed in the mean plantar pressure peak values in the HA of the left foot (*P*=.003) and right foot (*P*=.001) and in the LT of the left foot (*P*=.001) and right foot (*P*=.026). A significant increase was observed in the mean plantar pressure peak value in the MH position after fatigue in both the left and right feet (*P*=.001 and *P*=.017, respectively). The mean plantar pressure peak value in the right HA decreased by 32.6%, from 883.2 (366.8) gf to 595.1 (349.2) gf, and that in the left HA decreased by 31.9%, from 900.7 (396.2) gf to 612.8 (284.0) gf. The mean plantar pressure peak value in the right LT decreased by 31.1%, from 325.8 (210.6) gf to 223.7 (141.4) gf, and that in the left LT decreased by 33.9%, from 435.6 (162.0) gf to 287.8 (115.8) gf. The mean plantar pressure peak value in the right MH increased by 7.2%, from 1343.4 (385.7) gf to 1440.3 (406.8) gf, and that in the left MH increased by 11.0%, from 1265.5 (313.0) gf to 1404.9 (304.4) gf. The changes in the mean peak pressure in the M1, M5, and arch positions were not significant. However, the M1 and arch positions showed a change in the mean peak pressure in both the left and right feet. In contrast, we observed an increase in the mean peak pressure in the M5 position in the left and right feet of 12.5 (24.5) gf and 52.0 (79) gf, respectively. The details of the changes in the mean peak plantar pressures before and after fatigue exercise by sensor position via the wearable plantar pressure system are shown in Multimedia Appendix 1.

## Discussion

### Principal Findings and Comparison With Previous Works

The results of this study demonstrated that muscle fatigue in the gastrocnemius significantly affects the plantar pressure in the HA, LT, and MH regions in both the legs. In particular, the mean peak plantar pressure in the HA and LT regions significantly decreased and that in the MH regions significantly increased after 5 minutes of muscle fatigue exercise in the gastrocnemius compared with the value before the fatigue exercise. This finding was determined using the self-developed wearable foot plantar pressure system proposed in this research. Muscle fatigue exercise in this study induced muscle fatigue solely in the calf area, which corresponded with a significant decrease in the MDF of the sEMG of the gastrocnemius muscle but not in the MDF of the sEMG of the rectus femoris. As presented, sEMG is highly muscle-specific and effective for detecting localized muscle fatigue; it is not useful to detect muscle fatigue in deeper muscles like the tibialis posterior in the calf area [5]. Pressure sensors offer an alternative by measuring the fatigue in muscles through shifts in loading patterns, providing a broader and more accessible assessment.

The findings of this study align with existing literature, showing that muscle fatigue in the lower limb induces a significant decrease in the mean pressure in the HA area and a shift in plantar pressure from the forefoot to the hindfoot [21,23,30,31]. This could be due to individuals starting to adapt their plantar pressure pattern from the forefoot to the hindfoot to avoid overuse of the forefoot [23]. The literature also revealed a significant decrease in the first metatarsal region. Similar findings were observed in this study, but the results in the first and fifth metatarsals and arch regions were not statistically significant. This discrepancy could stem from differences in fatigue protocols, as more intensive activities, such as 30-minute runs, lead to greater pronation and increased medial midfoot loading, whereas walking shows no such midfoot differences [32-35]. Additionally, subject variability, including anatomical factors such as arch height and leg-length differences, may contribute to variance in medial midfoot pressure [30,36]. For instance, flat feet increase loading on the medial longitudinal arch, while high arches shift the load to the lateral edge [37].

In this study, a shift in loading from the forefoot region including the HA and LT to the hindfoot including the MH after 5 minutes of fatigue exercise results in gastrocnemius muscle fatigue. This finding could serve as an indicator for real-time monitoring of lower limb muscle fatigue, aiding in the prevention of injuries or falls in older adults and athletes. When a healthy person participates in sports such as badminton or running, muscle fatigue of the lower limb may be expected. These fatigued muscles can result in compromised reaction times, joint stability, and dynamic balance, thereby reducing impact absorption, which heightens the risk of injuries [21,38]. Athletes and coaches should monitor for fatigue alerts and ensure timely rest to recover muscle strength, preventing performance issues and injuries [39]. For older populations, particularly those at high risk of falls, such as patients with sarcopenia [40] or stroke [41], this system enables continuous home-based monitoring of plantar pressure patterns. A shift in plantar loading from the forefoot to the hindfoot could trigger alerts for rest to individuals or caretakers, minimizing fall risks associated with muscle fatigue.

Usability is a key factor in the adoption of digital health technologies such as the proposed wearable plantar pressure system [42]. Compared to sEMG, MMG, NIRS, or IMU wearable devices, its shoe-based form offers a user-centered design for daily wear, eliminating the need for users to remember and attach it correctly, thereby enhancing acceptance and compliance in home settings. A previous study highlighted the importance of face-to-face interactions with physicians in increasing their trust in digital health technologies [43]. Clinical or expert recommendations play a critical role in encouraging patients to adopt the device. The system’s ability to support effective remote patient management allows both users and experts to review and monitor past performance or clinical conditions, thus fostering engagement [44]. These features, combined with its user-friendly design and portability, enhance the system’s feasibility and scalability for home-based applications, promoting widespread adoption.

### Limitations

This study has some limitations. First, direct evidence for the development of muscle fatigue in the gastrocnemius could not be provided by this study, given that muscle force was not directly measured. Instead, the MDF of the sEMG signal, which shifted to a low frequency, was used as an indicator of muscle fatigue; this approach is a common research method for measuring muscle fatigue [45-47]. The decrease in MDF was likely caused by changes in the properties of the muscle fibers, such as decreased conduction velocity and increased muscle fiber recruitment [48]. The MDF of the sEMG measurement might also be affected by factors other than fatigue, such as the muscle fiber type [49] and motor unit firing rate [50]. However, this limitation was compensated by the RPE scale questionnaire. Second, the effects of fatigue were measured only in the rectus femoris and calf gastrocnemius muscles; the effect of upper leg fatigue on plantar pressure was ruled out. sEMG measurements of other calf muscles, such as the tibialis anterior, can provide insights into a particular calf muscle’s interaction with plantar pressure. The short test duration and limited wearing time (10‐20 min) in this study may not accurately represent real-life scenarios, necessitating extended testing to better simulate prolonged activities. In addition, wearable devices must adapt to various environments and wearing conditions, as factors such as daily wear and tear, temperature, humidity, flooring, and uneven terrain can impact sensor performance.

### Conclusions

The results of this study demonstrated a significant decrease in the mean plantar peak pressure in the HA and LT and an increase in the mean plantar peak pressure in the MH as an indicator of the onset of muscle fatigue in the gastrocnemius. This work is an exciting proof-of-concept outcome showing that muscle fatigue in the gastrocnemius can be detected via a wearable plantar pressure system. These findings can be used to further develop a wearable lower limb muscle fatigue monitoring system for minimizing injury risk in sports or during the daily activities of older adults.

## Supplementary material

10.2196/65578 Multimedia Appendix 1 Mean peak plantar pressures before and after fatigue exercise by sensor position via the wearable plantar pressure system system.

## Abbreviations

EMG: electromyography
HA: hallux
IMU: inertial measurement unit
LT: lesser toe
M1: first metatarsal
M5: fifth metatarsal
MDF: median frequency
MH: medial heel
MMG: mechanomyography
NIRS: near-infrared spectroscopy
RPE: rating of perceived exertion
sEMG: surface electromyography
