# Supplementary material for: Effectiveness of Using a Digital Wearable Plantar Pressure Device to Detect Muscle Fatigue: Within-Subject, Repeated Measures Experimental Design
Source: JMIR Hum Factors. 2025 Jan 7;12:e65578. doi: 10.2196/65578 (PMC11731697; doi:10.2196/65578)
Supplement: Multimedia Appendix 1 [file humanfactors-v12-e65578-s001.docx]

Multimedia Appendix 1.

Mean peak plantar pressures before and after fatigue exercise by sensor position via the wearable plantar pressure system

| Parameter | | The Wearable plantar pressure System – Clinical | | P value Pre–Post |
| --- | --- | --- | --- | --- |
|  |  | Mean Peak Pressure(gf) ± SD | |  |
|  |  | Pre | Post |  |
| Left Foot | Hallux (HA)^a^ | 900.7 ± 396.2 | 612.8 ± 284.0 | **0.003** |
|  | Lesser Toes (LT)^a^ | 435.6 ± 162.0 | 287.8 ± 115.8 | **0.001** |
|  | First Metatarsal (M1) | 601.3 ± 291.9 | 524.9 ± 289.7 | 0.091 |
|  | Fifth Metatarsal (M5) | 398.9 ± 117.2 | 411.4 ± 92.7 | 0.189 |
|  | ARCH | 292.0 ± 192.0 | 303.9 ± 201.0 | 0.189 |
|  | Medial Heel (MH)^a^ | 1265.5 ± 313.0 | 1404.9 ± 304.4 | **0.001** |
| Right Foot | Hallux (HA)^a^ | 883.2 ± 366.8 | 595.1 ± 349.2 | **0.001** |
|  | Lesser Toes (LT)^a^ | 325.8 ± 210.6 | 223.7 ± 141.4 | **0.026** |
|  | First Metatarsal (M1) | 659.9 ± 288.6 | 716.6 ± 520.5 | 0.304 |
|  | Fifth Metatarsal (M5) | 372.3 ± 120.6 | 424.3 ± 199.6 | 0.100 |
|  | ARCH | 267.9 ± 156.2 | 195.5 ± 95.4 | 0.064 |
|  | Medial Heel (MH)^a^ | 1343.4 ± 385.7 | 1440.3 ± 406.8 | **0.017** |
| ^a^ Significant difference (P < 0.05) | | | | |
